# External Validation of the PACTO Risk Score for Infective Endocarditis in Patients With Coagulase-Negative Staphylococcal Bacteremia: A Multicenter Cohort Study

**DOI:** 10.1093/ofid/ofag599

**Published:** 2026-09-23

**Authors:** Antonio Ramos-Martínez, Itziar Diego-Yagüe, Luis Eduardo López-Cortés, María Sánchez-Ledesma, Carmen Sáez-Béjar, Carmen Hidalgo-Tenorio, Marta Murga de la Fuente, Jesús Rodríguez-Baño, David Puertas-Miranda, Benedetta Varisco, Marianela Ciudad-Sañudo, Inmaculada López-Hernández, Jorge Calderón-Parra

**Affiliations:** Infectious Diseases Unit, Hospital Universitario Puerta de Hierro, Autonomous University of Madrid, Instituto Investigación Sanitaria Puerta de Hierro—Segovia de Arana (IDIPHSA), Majadahonda, Spain; Infectious Diseases Unit, Hospital Universitario Puerta de Hierro-Majadahonda, Instituto Investigación Sanitaria Puerta de Hierro—Segovia de Arana (IDIPHSA), Madrid, Spain; Unidad Clínica de Enfermedades Infecciosas y Microbiología, Hospital Universitario Virgen Macarena, Seville, Spain; Facultad de Medicina, Universidad de Sevilla, Seville, Spain; Instituto de Biomedicina de Sevilla (IBiS)/CSIC, Sevilla, Spain; CIBERINFEC, Instituto de Salud Carlos III, Madrid, Spain; Infectious Diseases Unit, Department of Internal Medicine, Complejo Asistencial Universitario de Salamanca, University of Salamanca, Instituto Investigación Biomédica de Salamanca, Salamanca, Spain; Infectious Diseases Unit, Hospital Universitario de la Princesa, Universidad Autónoma de Madrid, Instituto de Investigación Sanitaria de la Princesa (IIS-P), Madrid, Spain; Servicio de Enfermedades Infecciosas, Departamento de Medicina, Hospital Universitario Virgen de las Nieves, Granada, Instituto de Investigación Biosanitario de Granada (IBS-Granada), Universidad de Granada, Spain; Infectious Diseases Unit, Hospital Universitario Puerta de Hierro-Majadahonda, Instituto Investigación Sanitaria Puerta de Hierro—Segovia de Arana (IDIPHSA), Madrid, Spain; Unidad Clínica de Enfermedades Infecciosas y Microbiología, Hospital Universitario Virgen Macarena, Seville, Spain; Facultad de Medicina, Universidad de Sevilla, Seville, Spain; Instituto de Biomedicina de Sevilla (IBiS)/CSIC, Sevilla, Spain; CIBERINFEC, Instituto de Salud Carlos III, Madrid, Spain; Department of Internal Medicine, Complejo Asistencial Universitario de Salamanca, Spain; Department of Biomedical Sciences, Humanitas University, Milan, Italy; Infectious Diseases Unit, IRCCS Humanitas Research Hospital, Milan, Italy; Infectious Diseases Unit, Hospital Universitario de la Princesa, Instituto de Investigación Sanitaria de la Princesa (IIS-P), Madrid, Spain; Unidad Clínica de Enfermedades Infecciosas y Microbiología, Hospital Universitario Virgen Macarena, Seville, Spain; Facultad de Medicina, Universidad de Sevilla, Seville, Spain; Instituto de Biomedicina de Sevilla (IBiS)/CSIC, Sevilla, Spain; CIBERINFEC, Instituto de Salud Carlos III, Madrid, Spain; Infectious Diseases Unit, Hospital Universitario Puerta de Hierro-Majadahonda, Instituto Investigación Sanitaria Puerta de Hierro—Segovia de Arana (IDIPHSA), Madrid, Spain

**Keywords:** bacteremia, blood culture, coagulase-negative staphylococcus, endocarditis, heart valve prosthesis

## Abstract

**Background:**

Coagulase-negative staphylococci (CoNS) are a leading cause of prosthetic-valve infective endocarditis (IE) and an emerging cause of native-valve IE. Identifying patients with CoNS bacteremia at high risk of IE remains a significant clinical challenge. The PACTO score, derived from a single-center cohort, integrates four early-available predictors of IE.

**Methods:**

We conducted a multicenter retrospective cohort study of 302 adults with true CoNS bacteremia who underwent echocardiography at several Spanish hospitals between October 2016 and September 2025. IE was diagnosed according to the 2023 Duke-ISCVID criteria. The PACTO score assigns points for prosthetic valve or pacemaker (4), all blood culture bottles positive (2), community-acquired bacteremia (2), and continuous bacteremia (positive cultures 12–72 hours apart; 1).

**Results:**

Definite IE was diagnosed in 60 patients (19.9%). The PACTO score showed excellent discrimination (AUC 0.828; 95% CI .773–.883) and good calibration (Hosmer-Lemeshow *P* = .360). IE prevalence increased progressively across risk categories, from 1.6% (0–1 points) to 84.6% (>7 points). A score <2 points yielded a negative predictive value of 98.4% (sensitivity 98.3%).

**Conclusions:**

This multicenter external validation demonstrates that the PACTO score provides robust risk stratification for IE in patients with CoNS bacteremia, supporting safe deferral of echocardiography in low-risk patients and prompt imaging in high-risk individuals. Prospective validation is warranted.

## Teaser

The PACTO score accurately stratifies infective endocarditis risk in patients with coagulase-negative staphylococcal bacteremia. In this multicenter external validation, it demonstrated excellent discrimination and good calibration. A score below 2 points safely excludes endocarditis with a negative predictive value of 98.4%.

Coagulase-negative staphylococci (CoNS) are currently the second leading cause of infective endocarditis (IE) involving prosthetic valves and an emerging cause of IE involving native valves [1–3]. This rising incidence has been linked to the progressive aging of the population and the increased use of invasive diagnostic and therapeutic procedures [4]. CoNS-IE typically follows a subacute course with high rates of valve destruction, abscess formation, heart failure, surgical need, and mortality comparable to S. aureus IE [2–4]. However, most CoNS-positive blood cultures reflect milder infections like catheter-related bacteremia or mere contamination [5, 6]. Identifying patients at higher risk of developing IE and distinguishing true bacteremia from blood culture contamination remain significant clinical challenges [5, 6].

Several studies conducted in recent years have identified clinical and microbiological variables associated with the risk of developing IE in patients with bacteremia caused by *S. aureus*, *Enterococcus faecalis*, and non-β-hemolytic streptococci [7–12]. The risk of IE in patients with CoNS bacteremia has been much less studied; therefore, we lack externally validated tools to support clinical decision-making in this context [13–15].

Our group recently developed the PACTO score from a single-center cohort, integrating early-available predictors of IE in patients with CoNS bacteremia who underwent echocardiography [16].

We conducted a multicenter external validation of the PACTO score to assess IE risk and guide echocardiography decisions in patients with CoNS bacteremia [8, 11, 12, 17].

## METHODS

We conducted a multicenter retrospective cohort study to externally validate the PACTO risk score for infective endocarditis (IE) in patients with coagulase-negative staphylococcal (CoNS) bacteremia. Data came from the Spanish PROBAC cohort [15] and four hospitals in Granada, Salamanca, Madrid, and Majadahonda. The inclusion period spanned from October 2016 to September 2025.

### Study Population

#### Inclusion Criteria

Patients aged 18 years or older with true CoNS bacteremia who underwent echocardiographic (transthoracic and/or transesophageal) evaluation were eligible for inclusion.

#### Exclusion Criteria

We excluded patients with blood cultures deemed contaminated and those with polymicrobial bacteremia. In patients with several bacteremia episodes, only the first one was included in the analysis.

### Data Collection

The unified registry included comprehensive demographic, clinical, microbiological, echocardiographic, clinical management, and prognostic data. We assessed 30-day mortality following the onset of bacteremia. The study protocol was approved by the Institutional Review Board of Hospital Universitario Puerta de Hierro (Madrid, Spain; protocol number PI_29/23) and by the ethics committees of all participating hospitals. Given the observational nature of the study, informed consent was waived. All methods were conducted in accordance with relevant guidelines and regulations, and the study adhered to STROBE reporting guidelines.

### Definitions

#### True Bacteremia Versus Contamination

To ensure clinical significance of CoNS bloodstream infections, episodes were included only if they met pre-established microbiological and clinical criteria [18]. Blood cultures were processed according to national guidelines [19]. In the context of CoNS identification, positive blood cultures were considered true bacteremia if (1) the patient exhibited symptoms consistent with infection (axillary temperature ≥37.2°C, tachypnea (>20 bpm), tachycardia (>100 bpm), or hypotension (systolic blood pressure <90 mm Hg or diastolic blood pressure <60 mm Hg) and positive blood culture bottles from more than one separate set showed the same CoNS species with identical antibiogram patterns. Otherwise, positive blood cultures were classified as contaminants and excluded from analysis.

Infection-related mortality was defined as death occurring during the active bacteremic episode without an alternative explanation or in the setting of refractory sepsis attributable to the CoNS [20].

#### Bacteremia Classification

Bacteremia episodes were classified as community-acquired or healthcare-associated according to previously published criteria [21]. Continuous bacteremia was defined as positive blood cultures obtained between 12 and 72 hours after the initial blood culture despite appropriate antibiotic therapy. This definition was selected to align with clinical practice variability and Duke criteria [6, 22].

#### Infective Endocarditis Diagnosis

Only episodes meeting the Duke criteria for definite infective endocarditis at the time of diagnosis were included [6, 22]. Before the new Duke criteria were introduced [6], the diagnosis of IE was made by classifying CoNS as a non-typical pathogen [23]. There was no harmonization among the participating hospitals regarding the number of vials obtained. Diagnosis and treatment decisions were made by the local endocarditis team or expert clinicians at each participating center. All patients with CoNS bacteremia included in the study had undergone echocardiographic evaluation. There were no pre-established guidelines regarding the indications for echocardiography; consequently, the test was performed on patients in whom the likelihood of endocarditis was not considered clinically unlikely.

#### Other Clinical Definitions

Chronic renal failure was defined as a baseline glomerular filtration rate <60 mL/min per 1.73 m^2^ according to KDIGO criteria [24].

### Statistical Analysis

#### Risk Factor Identification

Continuous variables were expressed as medians with interquartile ranges (IQR) and categorical variables as absolute frequencies and percentages. Comparisons between patients with and without IE were performed using the Mann-Whitney U test for continuous variables and the chi-square or Fisher exact test for categorical variables, as appropriate.

Variables with *P*-values <.1 in the univariate analysis were included in a predictive multivariate logistic regression model based on their potential association with IE, taking into account the inclusion of 10 patients who met the dependent variable criteria (IE). Adjusted odds ratios (OR) with 95% confidence intervals (CI) were calculated. A two-tailed *P*-value <.05 was considered statistically significant. All statistical analyses were performed using SPSS software version 26.0 (IBM Corp., Armonk, NY, USA).

#### PACTO Score Validation

The PACTO score assigns points based on four variables: **P**rosthesis or pacemaker (4 points), **A**ll blood culture bottles positive (2 points), **C**ommunity acquisition (2 points), and con**T**inuous **O**ngoing bacteremia defined as positive cultures 12–72 hours apart (1 point). The total score ranges from 0 to 9 points [16].

We calculated the PACTO score for all patients in the validation cohort and evaluated its discriminative ability using receiver operating characteristic (ROC) curve analysis. The area under the ROC curve (AUC) with 95% CI was calculated to assess overall performance. For different score cutoffs, we determined sensitivity, specificity, positive predictive value (PPV), negative predictive value (NPV), positive likelihood ratio (LR+), and negative likelihood ratio (LR-). We stratified patients by PACTO score into risk categories (0–1, 2, 3–4, 5–7, and >7 points) and calculated the proportion of IE cases in each category.

Artificial intelligence tools were used in the preparation of this manuscript solely for language editing to improve clarity and readability. Claude (Anthropic) was also used to generate Figure 1 (calibration curve) from the study dataset. All AI-generated content was reviewed and verified by the authors, who take full responsibility for the accuracy and integrity of the work.

### Data Availability

The datasets used and analyzed during the current study are available from the corresponding author upon reasonable request.

### AI Assistance

Claude (Anthropic, San Francisco, CA, USA) was used for language editing and for generating Figure 1.

## RESULTS

### Characteristics of the Study Population

The prevalence of definite IE in the validation cohort was 19.9% (60/302), compared with 14.2% observed in the derivation cohort. Of the patients with IE, 35% (21/60) had native-valve IE, 51.7% (31/60) prosthetic endocarditis, and 13.3% (8/60) CIED-related IE. In the group of patients with IE in the validation cohort (n = 60), 50 (83.3%) underwent transthoracic echocardiography (TTE) and 40 (66.7%) underwent transesophageal echocardiography (TEE), with 30 (50.0%) requiring both imaging modalities for a definitive diagnosis. Conversely, among the non-IE patients (n = 242), TTE was the most frequent procedure, performed in 197 (81.4%) cases, while TEE and the combination of both techniques were utilized in 73 (30.1%) and 28 (11.5%) patients, respectively.

### Univariate Analysis

Table 1 summarizes the baseline characteristics, comorbidities, bacteremia characteristics, and clinical outcomes of patients with and without IE. Patients who developed IE were significantly older than those with bacteremia without IE (median age 75 years vs 70 years; *P* = .038). Community-acquired infection was more common among patients with IE (38%, 23 patients) compared with those without IE (22%, 52 patients; *P* = .007).

**Table 1.** Characteristics of Patients With CoNS Bacteremia According to Whether the Presence of IE

Variable	Total (n = 302)	IE (n = 60)	No IE (n = 242)	*P*
Age (years)	72 (62–79)	75 (68–81)	70 (59–79)	.038
Gender (female)	108 (35.8)	18 (30)	90 (37.2)	.298
Charlson Comorbidity Index	3 (1–7)	3 (1–7)	3 (2–6)	.763
Diabetes mellitus	79 (26.2)	21 (35)	58 (24)	.081
COPD	50 (16.6)	11 (18.3)	39 (16.1)	.679
Chronic renal failure	78 (25.8)	22 (36.7)	56 (23.2)	.032
Hemodialysis	14 (4.6)	3 (5)	11 (4.6)	1.000
Malignancy	57 (18.9)	13 (21.7)	44 (18.2)	.536
Solid organ transplant	26 (8.6)	5 (8.3)	21 (8.7)	.932
Liver cirrhosis	15 (5.0)	4 (6.8)	11 (4.5)	.509
Dementia	22 (7.3)	2 (3.3)	20 (8.3)	.269
Chronic heart failure	118 (39.1)	41 (68.3)	77 (31.8)	<.001
Prosthetic valve	63 (20.9)	31 (51.7)	32 (13.2)	<.001
Cardiac implantable device	33 (10.9)	14 (23.3)	19 (7.9)	.001
Community-acquired	75 (24.8)	23 (38.3)	52 (21.5)	.007
Healthcare-associated	50 (16.6)	11 (18.3)	39 (16.1)	.679
Nursing home facility	23 (7.6)	3 (5)	20 (8.3)	.687
Nosocomial	177 (58.6)	26 (43.3)	151 (62.4)	.007
Persistent bacteremia (12–72 h)	91 (30.1)	25 (41.7)	66 (27.3)	.029
*Staphylococcus epidermidis*	249 (82.5)	51 (85)	198 (81.8)	.562
*Staphylococcus hominis*	34 (11.3)	7 (11.7)	27 (11.2)	>.999
*Staphylococcus capitis*	3 (1.0)	0	3 (1.2)	>.999
*Staphylococcus haemolyticus*	9 (3.0)	0	9 (3.7)	.213
*Staphylococcus lugdunensis*	5 (1.7)	1 (1.7)	4 (1.7)	>.999
*Staphylococcus caprae*	2 (0.7)	1 (1.7)	1 (0.4)	.358
All blood culture bottles positive	191 (63.2)	49 (81.7)	142 (58.7)	<.001
Bacteremia-related mortality	50 (16.6)	17 (28.3)	33 (13.6)	.006
30-day all-cause mortality	93 (30.8)	25 (41.7)	68 (28.1)	.042

Quantitative variables are expressed as median (IQR) are compared by means of Mann-Whitney's U. IE: infective endocarditis. Qualitative variables are expressed as absolute number and (percentage) and compared by means of Chi-squared test (or Fisher exact test whenever necessary).

Abbreviations: IQR, interquartile range; CoNS, coagulase-negative staphylococci; IE, infective endocarditis; COPD, chronic obstructive pulmonary disease.

Chronic non-cardiovascular comorbidities did not differ between the two groups in most variables. However, chronic heart failure, the presence of prosthetic valves, and implantable cardiac devices were significantly more prevalent in patients who developed infective endocarditis. Chronic renal failure was most common in patients with IE (Table 1).

In relation to the microbiological distribution of the cohort, *Staphylococcus epidermidis* was the most frequently isolated species, accounting for 85% (n = 51) of cases of IE and 81.8% (n = 198) of bacteremia without IE, with no significant differences observed between the two groups (*P* = .562). Meanwhile, *Staphylococcus lugdunensis* had a low and similar incidence in both subgroups (1.7% in both groups; *P* = 1). Patients with IE more frequently had positive cultures in all blood culture bottles obtained initially (82%, 49 patients vs 59%, 142 patients; *P* < .001). Likewise, continuous bacteremia was more common in the IE group (42%, 25 patients vs 27%, 66 patients; *P* < .001).

Regarding clinical outcomes, mortality due to infection was significantly higher in patients with IE (17 patients, 28.3%) compared with patients without endocarditis (33 patients, 14%; *P* = .006). In addition, higher all-cause mortality at 30 days was observed among patients with IE (42%, 25 patients vs 28%, 68 patients; *P* = .042).

### Multivariate Analysis

The variables included in the multivariate analysis were age, chronic heart failure, implantable cardiac devices or valvular prostheses, community acquisition, positivity in all cultures obtained, and continuous bacteremia. Six variables were selected because 60 cases of IE were recorded. Chronic renal failure was not included due to concerns that it might be less discriminatory and because of the risk of collinearity, such as nosocomial acquisition.

The variables independently associated with the development of endocarditis were chronic heart failure (OR = 2.57; 95% CI 1.14–5.76), presence of implantable cardiac devices or valvular prostheses (OR = 6.62; 95% CI 3.04–14.45), community-acquired bacteremia (OR = 2.87; 95% CI 1.38–5.98), and positivity in all blood culture bottles (OR = 3.98; 95% CI 1.77–8.94). Age and continuous bacteremia did not reach statistical significance (Table 2).

**Table 2.** Multivariate Analysis of Variables Related to the Risk of IE in Patients With Bacteremia Due to CoNS

	β Coeficient	*P* Value	OR	95% Confidence Interval for OR	
				Lower	Upper
Age	0.005	0.693	0.995	0.971	1.020
Chronic heart failure	0.942	0.022	2.565	1.142	5.758
Prosthetic valve/intracardiac device	1.890	0.000	6.662	3.035	14.450
Community-acquired bacteremia	1.053	0.005	2.867	1.376	5.975
Persistent bacteremia (≥12–72 h)	0.516	0.153	1.676	0.825	3.403
All extracted blood culture bottles positive	1.380	0.001	3.975	1.767	8.943
Constant	−3.688	0.000	<.001		

Abbreviations: IE, infective endocarditis; CoNS, coagulase-negative staphylococci; OR, odds ratio.

### Validation of the PACTO Predictive Risk Score

The PACTO score showed good calibration (Hosmer-Lemeshow test, *P* = .360) and excellent discrimination (AUC 0.828; 95% CI .773–.883; Figure 1). Calibration of the PACTO score was assessed graphically and statistically. The calibration curve (Supplementary Figure 1) demonstrated close agreement between predicted and observed probabilities of IE across the full range of risk categories, with all five pre-specified score groups clustering near the line of perfect calibration. The score showed good discrimination and calibration both in *Staphylococcus epidermidis* bacteremias (AUC 0.809, 95% CI .747–.871, Hosmer-Lemeshow test *P* = .374) and non-*Staphylococcus epidermidis* bacteremias (AUC 0.944, 95% CI .877–1.000, Hosmer-Lemeshow test *P* = .926), with no significant differences between both AUCs (AUC difference 0.135, 95% CI [−0.44]-.227). The score retained good discrimination and calibration in patients without intracardiac prosthesis (AUC 0.767, 95% CI .663–.871, Hosmer-Lemeshow test *P* = .635), whereas discrimination was lower in patients with prosthesis (AUC 0.619, 95% CI .502–.741, Hosmer-Lemeshow test *P* = .250). This finding is expected, because prosthetic material is a major component of the score, and all patients in this subgroup are high-risk. This high-risk profile reduces the discriminatory contribution of the remaining predictors.

**Figure 1.**
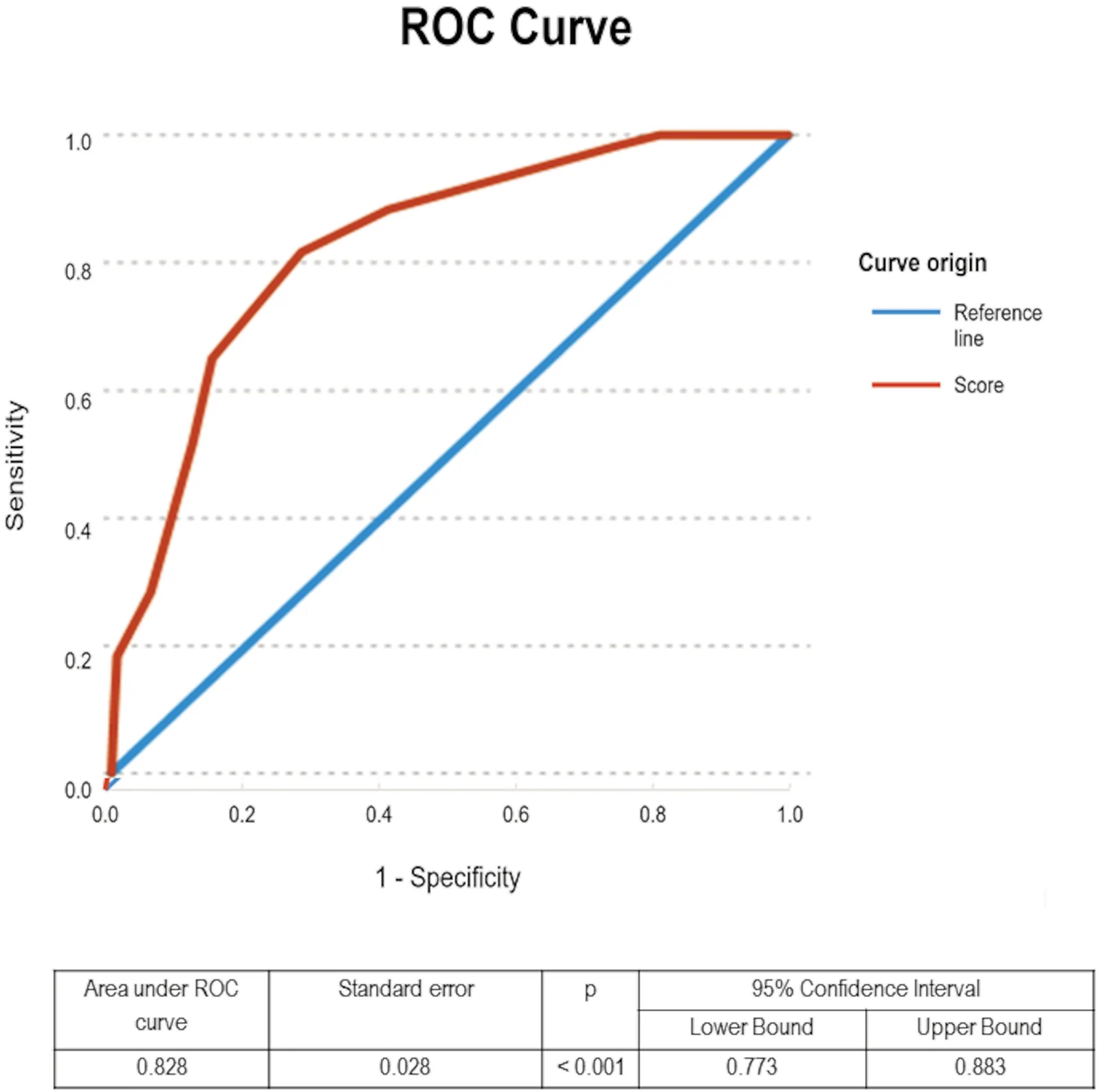
Receiver operating characteristic (ROC) curve analysis of the PACTO score for discriminating IE risk in patients with CoNS bacteremia.

The risk of IE in the validation cohort increased progressively with the number of points obtained on the score. The prevalence of IE increased significantly with higher scores: 1.6% for 0–1 points (1/62), 6.8% for 2 points (6/88), 18.2% for 3–4 points (14/77), 45.2% for 5–7 points (28/62), and 84.6% for more than 7 points (11/13) (Supplementary Table 1, Supplementary Figure 1). The one patient classified as low-risk and that did have IE was a 65-year-old male with prior diabetes mellitus and chronic cardiac failure (without prior severe known valvopathy or devices) that had a nosocomial bacteremia due to *Staphylococcus epidermidis*. He had persistent bacteremia for more than 48 hours (1 point in the score), which prompted the performance of a TEE that confirmed native valve IE.

The application of the cutoff in the validation cohort demonstrated that a score of less than 2 points (0 or 1) maintained a negative predictive value of 98.4% with a sensitivity of 98.3% (Table 3, Supplementary Table 2 and Supplementary Figure 2). The negative likelihood ratio of this cut-off was 0.06 A score of 2 points or higher had a positive predictive value of 24.6%.

**Table 3.** Operating Characteristics of the PACTO Risk Score Cut-off Point of for the Diagnosis of Infective Endocarditis in Patients With CoNS Bacteremia, Including Different Subgroups

Cut-off Point ≥2								
Population	Prevalence	AUC	S	E	NPV	PPV	LR−	LR+
Global	19.9% (60/302)	0.828	98.3%	25.2%	98.4%	24.6%	0.06	1.31
*S. epidermidis*	20.5% (51/249)	0.809	98.1%	23.7%	98.0%	24.9%	0.08	1.29
Non-S. *epidermidis*	16.9% (9/53)	0.944	100%	31.2%	100%	19.5%	0.00	1.45
Native valve	12.1% (29/239)	0.767	95.2%	30.7%	98.3%	15.8%	0.11	1.36
Prosthetic valve	49.2% (31/63)	0.619	100%	NC	NC	49.2%	NC	1
Community-acquired	30.7% (23/75)	0.800	100%	NC	NC	30.7%	NC	1
Healthcare-associated	22.0% (11/50)	0.729	100%	33.3%	100%	29.7%	0.00	1.50
Nosocomial	14.7% (26/177)	0.857	96.2%	31.8%	98.0%	19.5%	0.11	1.41

Abbreviations: AUC, area under the receiver operating characteristic curve; S, sensitivity; E, specificity; NPV, negative predictive value; PPV, positive predictive value; LR−, negative likelihood ratio; LR+, positive likelihood ratio; NC, non calculable.

## DISCUSSION

This multicenter external validation confirms that the PACTO score provides accurate risk stratification for IE in patients with CoNS bacteremia, demonstrating excellent discrimination (AUC 0.828; 95% CI .773–.883) and good calibration. Notably, the calibration curve showed close agreement between predicted and observed IE probabilities across all risk strata, from the very low-risk group (score 0–1, observed 1.6% vs predicted 2.4%) to the very high-risk group (score >7, observed 84.6% vs predicted 77.8%), confirming that the score not only discriminates between patients with and without IE, but also provides reliable absolute probability estimates that can directly inform clinical decision-making. The overall performance was comparable to that of the derivation cohort, where the AUC was slightly higher (0.87; 95% CI .77–.97), as is expected in external validation studies due to the well-described optimism of internally derived models.

The IE prevalence (19.9%) was higher than in the derivation cohort (14.2%) but falls within the 2%–23% range reported in the literature [13, 14, 16, 24, 25]. The species most frequently associated with IE was *S. epidermidis*, as has been shown in other studies [3, 25].

### Risk Factors for Infective Endocarditis in Patients With CoNS Bacteremia

All variables associated with IE in this study had previously been identified in other similar investigations involving *S. aureus*, *E. faecalis*, and non-β-hemolytic *Streptococcus* [8, 11, 12].

As observed in both the derivation and validation cohorts, the presence of a prosthetic valve or intracardiac device was the strongest predictor of IE [22]. Although CoNS are less virulent than *S. aureus*, their high ability to adhere to artificial surfaces through the production of various adhesins and the formation of biofilms favors infection of prosthetic valves and intracardiac devices [2, 26, 27]. These characteristics have been taken into account by the European Society of Cardiology (ESC) and the International Society for Cardiovascular Infectious Diseases (ISCVID) in classifying CoNS as typical pathogens causing endocarditis in patients with intracardiac prosthetic devices. Consequently, the threshold for CoNS bacteremia to meet major criteria has been lowered, reducing the requirements for both the number of positive bottles and the necessity of continuous bacteremia [6, 23].

Community-acquired CoNS bacteremia was also associated with IE in both cohorts, consistent with prior observations that hospital-acquired episodes are more frequently linked to catheter removal and carry lower IE risk [3, 4, 28, 29].

The association between positive results in all initial blood culture bottles and endocardial infection in both cohorts suggests the presence of a continuous intravascular infection, a typical feature of endocarditis [6, 23]. This finding is relevant because bacteremia with a high bacterial load, as occurs in IE, increases the probability that all bottles will be positive [30]. We should also note that continuous bacteremia is a distinctive feature of infective endocarditis [31]. Finally, positivity in all tubes is associated with true bacteremia rather than mere contamination [17, 32, 33].

In the multivariate analysis, continuous bacteremia was significantly associated with endocarditis in the derivation cohort, but not in the validation cohort [16]. Nevertheless, due to its clinical relevance, it was retained in the score evaluated in this study. Bacteremia in IE is typically continuous, allowing for its detection over time [34]. Zimmermann et al., in 466 CoNS bacteremia episodes, identified overlapping predictors—non-nosocomial acquisition, prosthetic material, and persistent bacteremia ≥48 hours—with an IE prevalence similar to ours (17% vs 19.9%) [29]. Three of these—non-nosocomial acquisition, prosthetic material, and continuous bacteremia—overlap directly with PACTO score variables, reinforcing their clinical relevance. The IE prevalence in their unselected bacteremia cohort (17%) was similar to ours (19.9%). Two key differences deserve mention: Zimmermann et al. used a persistence cutoff of ≥48 hours versus our broader 12–72-hour window, reflecting real-world variability in follow-up culture timing; and their model achieved a higher AUROC (0.949), partly explained by the inclusion of embolic events and prolonged symptom duration—variables only available later in the clinical course. The PACTO score's strength lies in its reliance exclusively on early, readily available data to guide echocardiography decisions within the first 24–72 hours of bacteremia.

Obtaining data on continuous bacteremia in CoNS infections is inherently challenging, as follow-up blood cultures are not routinely recommended in the absence of suspected intravascular device infection or an uncontrolled source [35]. The broad time window used in our study (12–72 hours) reflects the actual variability in blood culture practices at the participating centers, where follow-up cultures were often performed to meet a Duke criterion—which was in place for much of the study period—rather than to assess the microbiological response to treatment [23].

Age did not remain an independent predictor in the multivariate model but was retained given its established relevance in the derivation cohort.

### Clinical Applicability of PACTO Score

Using this score, we were able to classify patients into low (0–1 points, negative predictive value 98.4%) and moderate-to-high risk for IE (Supplementary Table 1). In fact, for pathogens more commonly associated with IE, such as *S. aureus* or *E. faecalis*, several scores were developed (VIRSTA, PREDICT, and NOVA); their cutoff points, which suggested against performing echocardiography, had negative predictive values similar to those shown in our validation study: 98.8%, 98.5%, and 95%, respectively [7, 8, 36].

A potential concern in our study is the verification bias arising from the exclusive inclusion of patients who underwent echocardiography. However, this methodological approach is consistent with the seminal derivation and validation studies of the VIRSTA, PREDICT, and NOVA scores, ensuring our results are comparable to the existing literature [7, 8, 10]. As shown in S. aureus bacteremia [37]. patients without echocardiography are lower-risk, so their inclusion would likely have maintained or increased the NPV. Therefore, their inclusion would likely have maintained or even increased the observed NPV of 98.4% for a PACTO score <2. By focusing on patients with imaging, we also mitigate the risk of “occult IE,” which has been independently associated with increased mortality when diagnostic tests are omitted.

Although not formally evaluated in this study, we recommend that patients scoring ≥4 points undergo TEE as the preferred initial imaging modality, particularly those with prosthetic valves, in whom TTE sensitivity is limited. For patients scoring 2–3 points, TTE may serve as a reasonable first approach, with TEE reserved for cases where clinical suspicion remains high or TTE findings are inconclusive.

### Limitations

This study has several relevant limitations. First, the retrospective nature and the potential verification bias—common to most IE prediction rules as discussed above—should be considered when interpreting our findings. Additionally, our study validates the score in patients with an echocardiogram performed, which may result in selection bias and a higher proportion of high-risk patients. However, this work is the first validation reported of the score and poses the basis for additional studies that validate the PACTO score in unselected patients, ideally in prospective cohorts. In addition, the fact that TEE was performed in a lower proportion of patients without endocarditis may have reduced the detection of cases of IE (possible false negatives). The definition of continuous bacteremia, based on positive blood cultures between 12 and 72 hours after the initial ones, constitutes a fairly broad time window, but one adapted to the actual variability in the timing of obtaining follow-up blood cultures among the participating centers. It cannot be ruled out that each hospital applied slightly different criteria to determine when to obtain these cultures. Additionally, prior antibiotic exposure was not systematically recorded, which may have influenced bacterial load and culture positivity. Additionally, although a subgroup analysis showed similar discrimination and calibration of the score in *Staphylococcus epidermidis* and other species, the number of episodes caused by non-Staphylococcus epidermidis species was low (n = 53), limiting the use of the score in other species. Finally, as the datasets were not specifically developed to develop or validate an IE-prediction score, some important variables, such as time to blood culture positivity, embolic events, or control blood culture availability, or the use of adjuntive imaging modalities were not available for analysis.

## CONCLUSIONS

This multicenter external validation demonstrates that the PACTO score accurately stratifies the risk of IE in patients with CoNS bacteremia, with results comparable to those of the derivation cohort. The score demonstrated good discriminative ability and calibration, and its high negative predictive value suggests that echocardiography could be reasonably deferred in patients at low risk. At the other end of the spectrum, the prevalence of IE exceeded 45% in patients scoring 5–7 points and reached 84.6% in those scoring above 7, underscoring the need for prompt echocardiographic evaluation—preferably TEE—in high-risk individuals, particularly those with prosthetic valves or intracardiac devices. These findings support the potential utility of the PACTO score as a practical tool to inform echocardiography decisions in patients with CoNS bacteremia, in an area where externally validated risk stratification tools are currently lacking.

## Supplementary Material

ofag599_Supplementary_Data

## Supplementary Data

Supplementary materials are available at *Open Forum Infectious Diseases* online. Consisting of data provided by the authors to benefit the reader, the posted materials are not copyedited and are the sole responsibility of the authors, so questions or comments should be addressed to the corresponding author.
