# Supplementary material for: External Validation of the PACTO Risk Score for Infective Endocarditis in Patients With Coagulase-Negative Staphylococcal Bacteremia: A Multicenter Cohort Study
Source: Open Forum Infect Dis. 2026 Sep 23;13(10):ofag599. doi: 10.1093/ofid/ofag599 (PMC13693480; doi:10.1093/ofid/ofag599)
Supplement: Supplementary file 1 [file ofag599_supplementary_data.docx]

**SUPPLEMENTARY MATERIAL**:

**Supplementary tables**:

**Supplementary table S1.** Risk stratification of infective endocarditis by PACTO score in patients with CoNS bacteremia

| Points | 0-1 | 2 | 3-4 | 5-7 | > 7 | Total |
| --- | --- | --- | --- | --- | --- | --- |
| IE | 1 (1.6%) | 6 (6.8%) | 14 (18.2%) | 28 (45.2%) | 11 (84.6%) | 60 (19.9%) |
| No IE | 61 | 82 | 63 | 34 | 2 | 242 |
| Total | 62 (20.5%) | 88 (29.1%) | 77 (25.5%) | 62 (20.5%) | 13 (4.3%) | 302 |

**Supplementary table S2.** Operating characteristics of the PACTO risk score for the diagnosis of infective endocarditis in patients with CoNS bacteremia

| Operating characteristics | | | | | | |
| --- | --- | --- | --- | --- | --- | --- |
| IE prevalence: 19.9% (60/302) | | | | | | |
| Points | Sensitivity | Specificity | NPV | PPV | LR- | LR+ |
| <1 | 100% | 19.0% | 100% | 23.5% | 0.00 | 1.23 |
| <2 | 98.3% | 25.2% | 98.4% | 24.6% | 0.06 | 1.31 |
| <3 | 88.3% | 59.1% | 95.3% | 34.9% | 0.19 | 2.16 |
| <4 | 81.7% | 71.9% | 94.1% | 41.9% | 0.25 | 2.91 |
| <5 | 65.0% | 85.1% | 90.0% | 54.2% | 0.41 | 4.36 |
| <6 | 51.7% | 88.8% | 86.7% | 63.0% | 0.54 | 4.62 |
| <7 | 28.3% | 94.2% | 84.1% | 54.8% | 0.76 | 4.88 |
| <8 | 18.3% | 99.2% | 83.0% | 85.0% | 0.82 | 22.9 |
| <9 | 10.0% | 99.6% | 81.7% | 86.1% | 0.904 | 25.0 |

ROC: Receiver operating characteristic. NPV: Negative predictive value. PPV: Positive predictive value. LR -: Negative likelihood ratio. LR+: Positive likelihood ratio

**Supplementary figures**:

**Supplementary figure S1**. Calibration curve of the PACTO score for the prediction of IEs in patients with CoNS bacteremia.

Colored circles represent the five pre-specified clinical risk categories (score 0–1, 2, 3–4, 5–7, and >7 points); marker size is proportional to the number of patients in each category. Grey circles represent decile-based groups of predicted probability, with the blue line showing the overall calibration trend. The dashed diagonal line indicates a good calibration (observed = predicted). Hosmer–Lemeshow test: χ²=5.48, degrees of freedom=5, p=0.360. IE: infective endocarditis. CoNS: coagulase-negative staphylococci.

Alt text figure 2. Figure 2 shows a calibration curve for the score regarding the prediction of endocarditis in patients with coagulase-negative staphylococcal bacteremia. This curve shows a good calibration (observed = predicted) with a Hosmer–Lemeshow test: χ²=5.48, degrees of freedom=5, p=0.360.

**Supplementary figure S2**. Distribution of the total study population and IE cases stratified by PACTO score

This figure shows a vertical bar histogram illustrating the percentage of patients with endocarditis across different score values. A progressive increase in the probability of endocarditis is observed as the score increases. The study population as a whole, however, shows high percentages of patients with medium or low scores on this score.
